# Is there a higher frequency of anal dysplasia and infection by human papillomavirus in Crohn's disease patients?

**DOI:** 10.1590/1414-431X2022e12141

**Published:** 2022-11-04

**Authors:** V.R. Guzela, C.W. Sobrado, S.R. Nadal, L.L. Villa, G.R. Mota, A.P. Gonçalves, C.R.M. Nadal, C.S.R. Nahas, S.C. Nahas

**Affiliations:** 1 Departamento de Gastroenterologia, Faculdade de Medicina, Universidade de São Paulo, São Paulo, SP, Brasil; 2 Instituto de Infectologia Emilio Ribas, São Paulo, SP, Brasil; 3 Departamento de Radiologia e Oncologia, Faculdade de Medicina, Universidade de São Paulo, São Paulo, SP, Brasil; 4 Instituto do Câncer do Estado de São Paulo, Hospital das Clínicas, Faculdade de Medicina, Universidade de São Paulo, São Paulo, SP, Brasil

**Keywords:** Anal cytology, Polymerase chain reaction, Dysplasia, Case-control, Human papillomavirus, Crohn's disease

## Abstract

The aim of this study was to compare the frequency of dysplasia and human papillomavirus (HPV) infection in the anal canal of patients with Crohn's disease (CD) with a control group and assess whether there is a correlation between use of immunosuppressants and anal manifestation of CD. Patients with CD and control individuals were submitted to anal cytology and material collection for polymerase chain reaction (PCR). The cytology was classified as normal, atypical squamous cells of undetermined significance (ASCUS), low-grade squamous intraepithelial lesion (LSIL), or high-grade (HSIL). PCR was considered positive or negative according to virus presence or absence. A total of 117 patients were included (54 in the control group and 63 in the CD group, being 32 without and 31 with immunosuppressants). ASCUS and LSIL were found in 25.9 and 22.2% of control patients and 28.6 and 39.7% of CD patients. HPV was identified in 14.8% of the control group and 27% of the CD group. In CD patients, HPV was found in 37.5 and 16.1% of those without and with immunosuppressants, respectively. Patients with perianal involvement had 15.6% of PCR positivity. There was no statistical difference in dysplasia and infection by HPV between the groups. Use of immunosuppressants did not influence the result, but anal manifestation was inversely proportional to viral detection.

## Introduction

Crohn's disease (CD) is an inflammatory bowel disease (IBD) that affects both genders with an average age of 30 years at the time of diagnosis (1). It is characterized by transmural lesions with granulomas that can compromise any part of the gastrointestinal tract (2,3). Perianal involvement of CD is present in 85% of patients during the course of the disease. However, the frequency of human papillomavirus (HPV) infection in the anal canal of this group is not well established nor is its role in the evolution of this condition (4,5).

The purpose of CD treatment is to induce and maintain disease remission. The most common strategy is to initially implement systemic-acting immunosuppressive medications. Despite having good long-term results, these drugs increase the risks of opportunistic infections and even some neoplasms (1,6).

Anal cancer is a rare disease in the general population accounting for only 0.4% of all new cancer cases in the United States. Its annual incidence (1.8/100.0 inhabitants) has increased in the last decade, with a five-year survival rate of 65.5% (7).

The most common histological type is squamous cell carcinoma, which has a high correlation with the presence of human papillomavirus (HPV) (8). In 84% of these tumors, it is possible to identify the virus, especially types 16 and 18. The other risk factors of HPV infection and squamous cell carcinoma are anal intercourse, high number of sexual partners, concomitant infection with *Chlamydia trachomatis* and human immunodeficiency virus (HIV), immunosuppression secondary to organ transplant therapy, chronic diseases, and smoking (9). Historically, the group with the highest risk is HIV-positive men who have sex with other men (10-12).

The relationship between HPV infection and disease and IBD, or more specifically, CD, has been discussed for more than 30 years due to the oncogenic potential of this interaction; however, the vast majority of publications assess the involvement of the cervix (13-18) or are case reports (19-22).

Regarding the anal canal, a study demonstrated that there is no difference in frequency of cytological alterations when comparing patients with CD to a control group (8.8 *vs* 2.6% of atypical cells of undetermined significance (ASCUS), respectively) (23). Another article that evaluated patients using anti-TNFα medications found 21.1% of HPV detection in the genital region (24). In these two publications, virus detection did not differ between CD and controls, but the individuals of the first group presented a greater presence of oncogenic high-risk HPV (25,26). So far, the relationship between CD and HPV remains controversial, therefore this article aimed to evaluate the frequency of anal dysplasia and HPV infection in this population to understand whether factors like immunosuppressive medications and anal disease may affect this response.

## Material and Methods

### Design and groups

The institutional review board of the University of São Paulo approved the study and all the participants provided informed consent. This was a cross-sectional, single-center study that included patients with CD and individuals without IBD, called controls (CT), matched by age and sex. All the individuals were patients at Hospital das Clínicas, Faculdade de Medicina (Brazil) and were recruited from 2016 to 2019.

The inclusion criteria were sexually active individuals, between 18 and 70 years old. Exclusion criteria were pregnancy, patients with previous or present diagnosis of HPV lesions or anal cancer, and patients who received HPV vaccines. In the control group, use of immunosuppressants and immunocompromising conditions such as HIV were also exclusion criteria. In the CD group, use of immunosuppressants was considered when the patient was receiving corticosteroids (prednisone, dose equal to or greater than 10 mg/day or equivalent), immunomodulators such as azathioprine (dose equal to or greater than 1 mg·kg^-1^·day^-1^ of body weight), or biological medicine (infliximab, adalimumab, certolizumab, vedolizumab, or ustequinumab at any dose at the time of the exam or up to 90 days before).

### Methodology

All invited individuals completed a questionnaire about social data, medical history, lifestyle, and sexual habits. Other information was completed based on medical records, as the period of IBD's evolution. To evaluate the activity of CD, the Harvey-Bradshaw score was calculated during the interview and the last level of C-reactive protein was considered. Hemoglobin level was documented in order to establish the general health status of the patients with CD. After this procedure, the patient underwent a proctological examination to verify the presence of suspect HPV lesion.

Patients underwent anal cytologic collection using two brushes (27) to make two smear slides preserved in 70% alcohol. A third brush was used to collect material for conditioning in liquid and subsequent molecular analysis for the presence of HPV and viral type. The brushes were inserted 4 cm up the anal verge, without lubricant, and then withdrawn with spiral motion.

The cytology results were based on the Bethesda classification (28) after being interpreted by two pathologists as normal, atypical squamous cells of undetermined significance (ASCUS), low-grade squamous intraepithelial lesion (LSIL), high-grade intraepithelial lesion (HSIL), or squamous cell carcinoma. In defining the results, the highest risk classification was considered the “cytological conclusion”. This method has a sensitivity of 88% and a specificity of 42.7% (29) when two samples are obtained. Another parameter used in this study is based on cytology and named “abnormal cytology”, defined as present when the cytological conclusion is ASCUS, LSIL, HSIL, or carcinoma.

The third sample deposited in the preserving liquid was submitted to polymerase chain reaction (PCR) followed by hybridization with the use of Linear Array^®^ kit (Roche Molecular Systems, USA), which allows identification of 37 different types of HPV and has 96% sensitivity and 99% specificity.

### Statistical analysis

Statistical analysis was performed using IBM SPSS^®^ 25 (IBM, USA). To test the hypothesis, Kruskal Wallis test for independent samples and Fisher's exact test were applied. All tests took into account a bidirectional α error of 0.05 and a 95% confidence interval (CI). For a test power of 80% and a significance level of 5%, the minimum required number of participants in each group was 24, based on studies of HIV prevalence.

## Results

**Figure 1:**
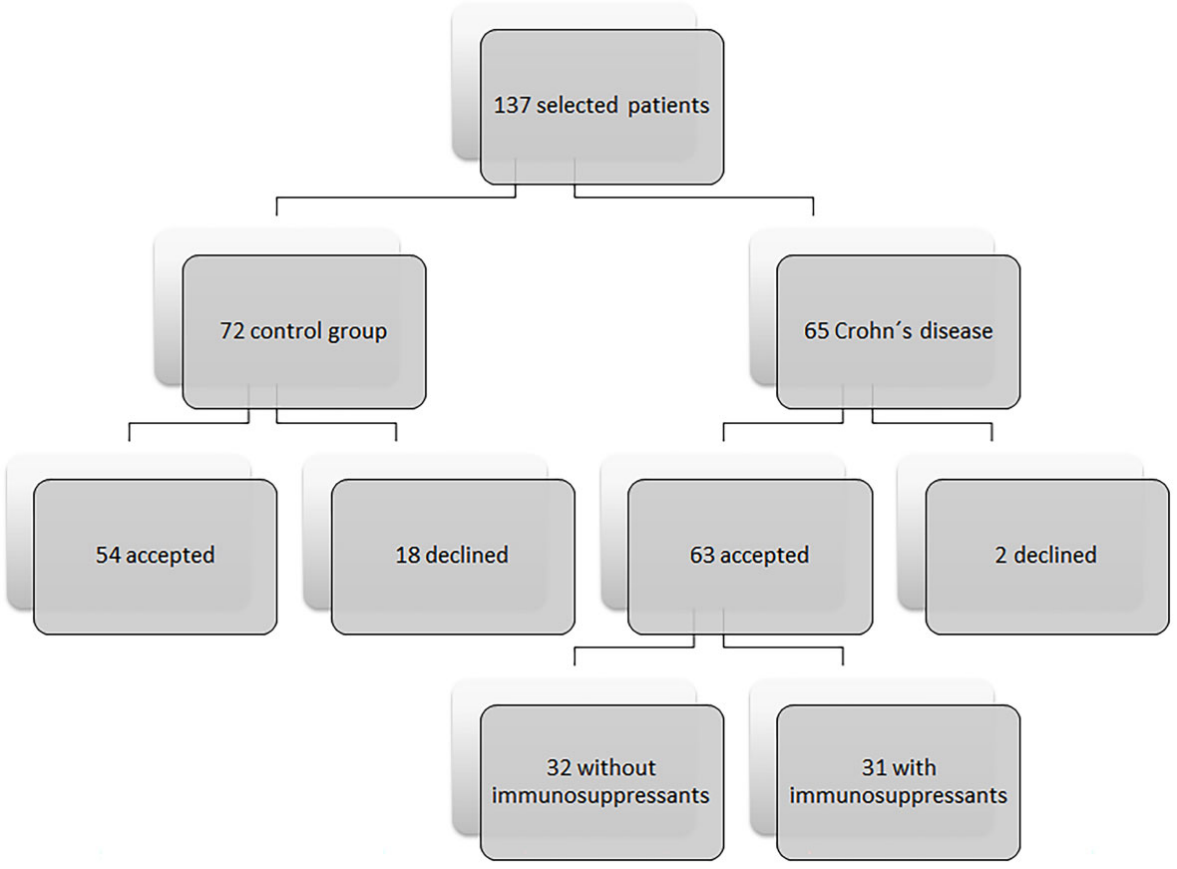
Flow diagram outlining group composition.

The recruitment flow of patients that consented to participate is illustrated in Figure 1.

In Table 1, the characteristics of both groups are described.

**Table 1:** Descriptive characteristics according of the control and Crohn's disease groups.

Variables	Control	Crohn’s disease	P-value
N	54	63	
Age, average (years)	44	43	0.844
Age at first intercourse, average (years)	17	19	0.004
Sexual partners (n)	5	3	0.005
Women (%)	53.7	52.4	0.954
Ethnicity (%)			
White	81.5	85.7	
Black	16.7	12.7	0.638
Asian	0	1.6	
Hispanic	1.8	0	
Marital status (%)			
Single	33.3	50.8	
Married	57.7	46.0	0.046
Divorced	13.3	3.2	
Schooling (%)			
Illiterate	1.9	1.6	
Elementary school	59.6	36.7	0.064
High school	34.6	51.7	
College or higher degrees	3.8	10.0	
Smoking (%)			
Positive	5.6	11.1	0.337
Alcohol intake (%)			
Positive	20.4	11.1	0.203
History of sexual disease (%)			
Positive	1.9	4.8	0.623
Proctological examination (%)			
Normal	20.4	28.6	
Fistulas	7.4	25.4	
Ulcers	0	7.9	
Skin tags	5.6	15.9	<0.001
Hemorrhoids	55.6	6.3	
Fissures	9.3	4.8	
Stenosis	1.9	11.1	

The Kruskal Wallis test for quantitative variables and Fisher's exact test for qualitative tests were used for statistical analyses.

In the CD group, 50.8% of the individuals had present or past anal inflammatory impairment and 36.5% of these patients had fistulizing condition. The laboratory and clinical parameters of the CD subgroups according to the use of immunosuppressants are described in Table 2.

**Table 2:** Laboratory and clinical parameters of the Crohn's disease subgroups according to the use of immunosuppressants.

	Without immunosuppressants	With immunosuppressants	P-value
Mean hemoglobin level (g/dL)	13.1	12.4	0.104
Mean C-reactive protein level (mg/dL)	13.6	11.1	0.827
Mean duration of Crohńs disease (years)	10	12	0.247
Mean Harvey-Bradshaw index	5	8	0.119

Data are reported as means. The Kruskal-Wallis test for independent samples was used for statistical analyses.

Table 3 describes the cytological conclusion and the PCR results comparing the CD and CT groups revealed that there are no statistical differences between them. HSIL was not found in any group.

**Table 3:** Cytological conclusion and molecular analysis results for the control and Crohn's disease groups.

	Control		Crohn’s disease		P-value
	N	%	N	%	
Cytology					
Normal	20	37.0	16	25.4	
Inadequate	8	14.8	4	6.3	
ASCUS	14	25.9	18	28.6	0.105
LSIL	12	22.2	25	39.7	
HSIL	0	0.0	0	0.0	
Molecular analysis					
Negative	45	83.3	46	73.0	
Positive	8	14.8	17	27.0	0.143
Inadequate	1	1.9	0	0.0	

Fisher's exact test was used for statistical analyses. ASCUS: atypical squamous cells of undetermined significance; LSIL: low-grade squamous intraepithelial lesion; HSIL: high-grade squamous intraepithelial lesion.

Table 4 shows the results of PCR in the CD subgroups according to the use of immunosuppressants. It also includes the results of detection of oncogenic high-risk types and specific type 16, which the first have a higher prevalence than the former, but not exactly dependent on the presence of type 16 presence.

**Table 4:** Human papillomavirus detection in the Crohn's disease subgroups according to the use of immunosuppressants.

	Without immunosuppressants		With immunosuppressants		Control		P-value
	N	%	N	%	N	%	
HPV							
Positive	12	37.5	5	16.1	8	14.8	0.064
Negative	20	62.5	26	83.9	45	83.3	
High risk							
Positive	8	66.7	3	60.0	7	87.5	0.600
Negative	4	33.3	2	40.0	1	12.5	
Type 16							
Positive	3	25.0	2	40.0	2	25	0.850
Negative	9	75.0	3	60.0	6	75	

Fisher’s exact test was used for statistical analyses. HPV: human papillomavirus.

Twenty different viral types were found (8 of them of oncogenic high risk), with some individuals presenting from 1 to 5 types in the same sample. The most frequent was 16 and the second most frequent was 58, both being oncogenic high-risk types.

In patients with anal involvement of the inflammatory disease, 15.6% were positive for HPV, whereas in patients without previous or current anal inflammatory activity, the frequency of HPV was 38.7% (P=0.05).

Table 5 shows the PCR results according to absence/presence of anal impairment and that the general HPV positivity was different between the subgroups.

**Table 5:** Human papillomavirus (HPV) detection in the Crohn's disease group according to anal manifestation.

	No anal evolvement		Anal evolvement		P-value
	N	%	N	%	
HPV					
Positive	12	38.7	5	15.6	0.050
Negative	19	61.3	27	84.4	
High risk					
Positive	6	50.0	5	100.0	0.102
Negative	6	50.0	0	0	
Type 16					
Positive	2	16.7	3	60.0	0.117
Negative	10	83.3	2	40.0	

Fisher’s exact test was used for statistical analyses.

## Discussion

In the control group, there was a greater refusal to participate in the study, which was attributed to the method still being met with greater rejection, especially in patients less used to medical procedures. The CT and CD groups had similar characteristics in almost all the social and economic aspects. There was a statistically significant difference in age at first sexual intercourse and number of different sexual partners, with patients of the CT group revealing a greater sexual activity compared to CD. Information bias may have occurred in both groups, since answering questions about sexual habits may be embarrassing and is influenced by social judgment.

The findings of the physical exam also differed between groups. Controls showed a higher frequency of hemorrhoidal disease and CD patients had more typical Crohn's disease manifestations. In the CD group, patients were divided according to whether or not they were using immunosuppressants. The clinical and laboratory parameters were similar, indicating that the groups were homogeneous populations, decreasing the impact of possible confounding factors for the risk of opportunistic infections.

The frequency of dysplasia secondary to HPV assessed using smear anal cytology was not different between CT and CD groups on cytological conclusion, and the higher frequency found for the CD group revealed a tendency for dysplastic changes in the anal canal. HSIL was not found in any individual, suggesting either a bias on the cytopathologist's interpretation or just the absence of advanced dysplasia in this population.

One article reported altered cytology in 48.4% of the patients with CD (25), while another one found ASCUS in 8.8% of IBD patients (23). It is believed that the higher detection of alterations in this study can be attributed to the use of two brushes for collecting anal cytology and making two smears, while the previous publications used a single swab to obtain the samples, which were stored and analyzed in the preservation liquid. A Brazilian publication had already demonstrated that collection with two brushes has sensitivity of 88% compared to 69% when only one brush is used (27).

Virus detection by PCR was also not different between groups, but it had a lower prevalence compared to abnormal cytology. A similar study compared IBD with a control group and also failed to find statistically significant differences (8.8 and 2.6%, respectively, P=0.1), despite noticing a trend towards higher frequency of altered cytology in the IBD group (23).

Previous publications (23-26) reported frequencies of 1.8, 21.1, 89.1, and 34% of HPV detection in the anal region of patients with IBD. The values are very different among themselves and from the results of this study, but these studies had different methodologies. One of them used Digene Hybrid Capture 2 (HC2) (Qiagen, Spain) to detect high-risk HPV in the same sample sent for cytology and identified HPV prevalence of 1.85%. The second study collected a swab from 222 patients with psoriasis or IBD from the anal, penile, or vulvar and cervical regions using the same HC2 kit (Qiagen, USA) for HPV detection. The third study, in addition to evaluating cytology, also conducted hybridization analysis and found 37 types of HPV (Bio-Rad) from the same material (46 IBD patients). The last study collected tissue samples prior to colonoscopies and viral detection was done by the INNO-LiPA HPV method (Fujirebio, Japan) in 101 patients with IBD. In this research, the Linear Array^®^ method was used, which is considered one of the most sensitive and specific methods.

When the CD subgroups with and without use of immunosuppressants were evaluated, similar results were found between them and when compared to the CT group. Consequently, the variable “use of immunosuppressants” did not seem to interfere in the frequency of HPV infections in the anal canal in the samples evaluated, contrary to what might be expected by the behavior of the virus in patients immunosuppressed by other etiologies (HIV or solid-organ transplant). Although there were no statistical differences between the groups, there was a tendency for higher prevalence especially in CD without immunosuppressants. Other studies also did not find positive relationships between prevalence of anal HPV and immunosuppression secondary to IBD therapy (23-25). However, one study found that immunosuppression was an independent risk factor for HPV presence in anal canal samples (OR=5.31; 95%CI: 1.94-14.59) (26).

Among PCR-positive patients, the vast majority had high-risk HPV regardless of the group or subgroup. More than 70% of the individuals included in the study were high-risk-positive, while previous data reported frequency of 13.8 to 30% (24,26). A previous study mentioned frequency of 65.2% for type 16 (25), but the present study found 28% of HPV 16 in the CD group. These findings suggested that in the present sample, there was a lower prevalence of type 16 and greater variability among other oncogenic high-risk types. Twenty different types of HPV were found, of which 8 were classified as oncogenic high-risk HPV and some individuals presented 5 different types concomitantly. The second most frequent type was HPV 58. The use of Linear Array^®^ allowed the identification of viral types that are not commonly detected by other molecular methods usually applied.

Anal manifestation of CD was not related to dysplastic cytological findings, since ASCUS and LSIL had similar prevalence in the CD group without anal disease. This suggests that, despite the difficulty in collecting anal brush samples or presence of local inflammation, the results of the two populations may be comparable. However, PCR revealed a statistically significant difference between patients with and without anal involvement, being higher in the former group. This divergent data can be attributed to a possible lower sexual exposure of those with perineal disease, a factor that interferes negatively in the quality of life, as reported previously (29). About 35 to 58% of patients with IBD have some degree of embarrassment in relation to sexual behavior and 73% of those with CD have body image disorders, often influenced by surgical history. Another explanation could be the unregulated local immunological response leading to a pro-inflammatory state that can contribute to an increase in local protection against the action of HPV. Various mechanisms such as altered lymphocyte activity, cross-response to antigens, and amplification of the TNFα-mediated response among others are known in IBD and may explain this finding (30). However, immunosuppressants did not seem to alter the prevalence in this study, since all patients taking immunosuppressants who had anal disease had negative PCR. Patients without this therapy but with anal involvement had a higher frequency of HPV 16.

Most reference articles on this topic that investigated viral action or presence of HPV were carried out in patients diagnosed with IBD, not being specifically designed for CD, and considered the use of immunosuppressants and anal manifestation.

Study limitations include the cross-sectional design and the possible information bias, which may compromise the extrapolation of data. Patients with inadequate cytology or PCR, as seen in Table 3, were invited for a second exam, but there was no adherence, which affected the overall evaluation of the study and resulted in sample loss, probably due to discomfort during the exam. These individuals were not included in the stratified analysis and it is important to describe this finding to demonstrate that the two-brush method reduces the frequency of lost samples and the use of two different methods of analysis allows for better evaluation.

In conclusion, this study did not find differences in cytological alterations and HPV presence in the anal canal between CD patients and a control group.
